# Real-world disease burden and planned treatment optimization after MANAGE-PD implementation in Germany: a cross-sectional study

**DOI:** 10.1186/s42466-025-00383-2

**Published:** 2025-05-12

**Authors:** Martin Südmeyer, David J. Pedrosa, Frank Siebecker, Carolin Arlt, Jaakko Kopra, Wolfgang H. Jost

**Affiliations:** 1 Department of Neurology, Ernst-Von-Bergmann Klinikum, Charlottenstr. 72, 14467 Potsdam, Germany; 2 Department of Neurology, Medical Faculty, University Düsseldorf, Moorenstr. 5, 40225 Düsseldorf, Germany; 3 Department of Neurology, Philipps-University Marburg, Baldingerstr., 35032 Marburg, Germany; 4 Praxis Neurologie, Mühlenstr. 14, 48291 Telgte, Germany; 5 AbbVie GmbH, Lembückgasse 61, 1230 Vienna, Austria; 6 AbbVie Deutschland GmbH & Co. KG, Mainzerstr. 81, 65189 Wiesbaden, Germany; 7 Parkinson-Klinik Ortenau, Kreuzbergstr. 12, 77709 Wolfach, Germany

**Keywords:** Parkinson’s disease, MANAGE-PD, Disease burden, Device-aided therapy, Clinical decision making

## Abstract

**Background:**

In Germany, the approach to treatment optimization for patients with advanced Parkinson's disease (PD) is considered somewhat conservative. The MANAGE-PD tool (www.managepd.eu) was developed to help identify patients with advanced PD and to facilitate treatment decision making and appropriate allocation of patients to device-aided therapies (DAT). This prospective, non-interventional study aimed to investigate the real-world disease burden of PD and treatment optimization after MANAGE-PD implementation.

**Methods:**

Adult PD patients (N = 278) visited specialist clinics and neurologist’s practices in Germany in 2022. Disease burden was assessed using the Unified PD rating scale (UPDRS parts II-IV), the non-motor symptoms scale (NMSS) and the 8-item Parkinson’s disease Questionnaire (PDQ-8). Data on planned treatment changes were collected. Data were analyzed by disease control categories according to the MANAGE-PD tool.

**Results:**

Mean scores for motor and non-motor symptoms, quality of life, and comorbidity burden were worse in patients with lower disease control measured by MANAGE-PD. For 52.8% of patients in Category 2 (inadequately controlled—might benefit from oral optimization), no change in oral treatment was planned. No change in oral treatment and no DAT initiation was planned for 37.9% and 65.0% of patients in Category 3 (inadequately controlled—might benefit from DAT). Patient refusal and needing more time to decide were the most common reasons for not making treatment changes.

**Conclusions:**

This study supports the validity of MANAGE-PD by showing its high association with disease burden and emphasizes the importance of timely provision of necessary information to enable informed decisions about treatment optimization.

**Supplementary Information:**

The online version contains supplementary material available at 10.1186/s42466-025-00383-2.

## Background

Parkinson’s disease (PD) is a progressive neurodegenerative disorder characterized by a variety of motor and non-motor symptoms. PD diagnosis has a significant impact on a patient’s life. In addition to the cardinal motor symptoms of tremor, rigidity, and bradykinesia, many patients experience non-motor symptoms such as cognitive dysfunction [18, 30], mood disorders [20], sleep disorders [5, 7], fatigue [23], and autonomic symptoms [17].

The treatment of PD initially involves oral dopaminergic medication such as levodopa or dopamine agonists, as well as monoamine oxidase (MAO) inhibitors. However, the effectiveness of oral medications typically diminishes after a few years of treatment, and it is recommended to consider device aided treatment (DAT) options for patients with inadequately controlled symptoms (Höglinger G., Trenkwalder C. et al., 2023).

In 2018, the leading experts in the field developed criteria for advanced PD and eligibility for DAT using the Delphi method [3, 4]. Based on these criteria, the Making Informed Decisions to Aid Timely Management of Parkinson’s Disease (MANAGE-PD) tool was created. The goal of this tool is to assist physicians in identifying advanced PD patients who might be inadequately controlled with current medication and might benefit from current therapy optimization or DAT implementation. The tool categorizes patients into three categories of disease control [1]. The MANAGE-PD tool has been validated [1, 2] and is registered as a Class I medical device. It has been freely available in Germany since 2020 online (www.managepd.eu) and as a paper-based version. Our previous publication investigated the real-world use and concordance of the MANAGE-PD tool outcomes across multiple specialist clinics and generalist neurology practices in Germany (Südmeyer et al. [29]). The validity and usability of a modified patient version of the MANAGE-PD Sect. 1 (“Parkinson Check”) was also investigated. Our results indicated high concordance between the tool outcomes and the physicians’ responses, especially in specialist clinics. The concordance between the physicians’ and patients’ responses was also remarkably high, justifying a wider use of the “Parkinson Check”.

Despite the established validity of MANAGE-PD, there is limited evidence on its real-world impact. Especially, prospective research on MANAGE-PD is lacking. In the above-mentioned prior work, we showed that the majority of PD patients were inadequately controlled, according to the tool’s categorization. Consequently, patients might benefit from a more objective treatment management. The aim of the current study was therefore to go one step further and assess the clinical characteristics and disease burden of PD patients based on the tool’s categories, as well as their planned treatment optimization following the implementation of MANAGE-PD.

## Methods

### Study design and study population

In this prospective, multicenter study, we conducted an observational, cross-sectional investigation on adult patients diagnosed with Parkinson's disease (PD) according to the Movement Disorder Society diagnostic criteria [21]. The recruitment of participants took place in specialized clinics (n = 10) and neurologists' practices (n = 9) in Germany from January 2022 to September 2022. We included PD patients currently undergoing oral levodopa therapy, but excluded those who had previously received or were currently receiving DAT treatment. All physicians involved followed the relevant legal and regulatory requirements. The study was conducted in accordance with the most recent version of the Declaration of Helsinki. The ethics committee of Landesärztekammer Brandenburg approved the study under the reference number 2021–2188-BO-ff. Prior to participation, all patients provided written informed consent.

### Data collection

Demographic and clinical characteristics were collected from each patient during a single routine visit. Physicians assessed disease severity and burden with the original Unified Parkinson’s Diseases Rating Scale (UPDRS parts II-IV) [10], the modified Hoehn and Yahr stage [14], and the Non-Motor Symptoms Scale for Parkinson’s disease (NMSS) questionnaire [8]. Additionally, patients completed the 8-item Parkinson’s disease Questionnaire (PDQ-8) [16]. Higher scores indicate a higher disease burden/impairment of quality of life.

Initially, physicians rated patients’ disease control followed by assessments using the MANAGE-PD tool [1], [29]. Disease control categories included “Adequately controlled on current oral therapy” (Category 1), “Inadequately controlled under current oral therapy—might benefit from further oral optimization” (Category 2), and “Inadequately controlled under current oral therapy—might benefit from DAT” (Category 3) [1, 2]. Additionally, data on planned changes in oral treatment as well as DAT initiation were collected.

### Statistical analysis

Descriptive analysis was performed on all data from patients who met the selection criteria as well as stratified analysis based on disease control categories according to the MANAGE-PD tool (Categories 1–3). To investigate symptoms (according to MANAGE-PD tool Sect. 2) that might be associated with patient’s eligibility for DAT (physician’s assessment), a logistic regression model was performed for patients in MANAGE-PD tool Category 2 and 3. A forward selection procedure (significance level for entering a variable into the model = 0.05) was applied to identify the most influential variables and to reduce multicollinearity.

All statistical analyses were conducted with SAS® 9.4 (SAS Institute, Cary, NC, USA).

## Results

### Patient characteristics

In total, 287 patients were enrolled from 19 sites [29]. 278 patients (96.9%) fulfilled the selection criteria and were included in the analyses. Mean age was 70.8 years, and almost two-thirds of the patients were male (Table 1).

**Table 1:** Patient characteristics and comorbidities

	Disease control categorization by MANAGE-PD tool			Total (N = 278)
	Category 1 (n = 50)	Category 2 (n = 125)	Category 3 (n = 103)	
Age [years], mean ± SD	69.8 ± 7.6	71.3 ± 10.5	70.6 ± 10.8	70.8 ± 10.1
*Sex*				
Male, n (%)	40 (80.0)	76 (60.8)	64 (62.1)	180 (64.7)
Female, n (%)	10 (20.0)	49 (39.2)	39 (37.9)	98 (35.3)
At least one comorbidity*, n (%)	36 (72.0)	111 (88.8)	93 (90.3)	240 (86.3)
Orthostatism	4 (8.0)	9 (7.2)	15 (14.6)	28 (10.1)
Polyneuropathy/Neuropathy	1 (2.0)	12 (9.6)	11 (10.7)	24 (8.6)
Depression	5 (10.0)	26 (20.8)	28 (27.2)	59 (21.2)
Cognitive dysfunction	4 (8.0)	22 (17.6)	25 (24.3)	51 (18.3)
Sleep disorders	10 (20.0)	32 (25.6)	31 (30.1)	73 (26.3)
Fatigue	2 (4.0)	6 (4.8)	13 (12.6)	21 (7.6)
Hypertension	18 (36.0)	43 (34.4)	46 (44.7)	107 (38.5)

^1^Physician’s judgement of the patient before implementing the MANAGE PD tool, Category 1: Currently well controlled, Category 2: Inadequately controlled – might benefit from further oral optimization, Category 3: Inadequately controlled – might benefit from DAT, N/n: number of patients, SD: standard deviation

^*^Includes PD and/or non-PD related comorbidities

Average time since diagnosis was 7.4 years. According to the MANAGE-PD tool, 50 patients (18%) were currently well controlled (Category 1), 125 patients (45%) were inadequately controlled but might benefit from further oral medication optimization (Category 2), and 103 patients (37%) were inadequately controlled and might benefit from DAT (Category 3). Patient characteristics are described in detail elsewhere [29]. In general, patients in Category 2 and 3 showed similar characteristics. Around 70% of the patients in Category 1 and around 90% of the patients in Category 2 and Category 3 suffered from at least one (PD and/or non-PD related) comorbidity (Table 1). For almost all PD-related comorbidities, the proportion of affected patients was lowest in Category 1 and highest in Category 3. PD- and non-PD related comorbidities are shown in Additional file, Table 1. Most patients reported having at least one comorbidity (86.3%) and common comorbidities included hypertension (38.5%), sleep disorders (26.3%), cardiac abnormalities or cardiovascular disease (23.0%), and depression (21.2%). While only one patient (2%) in Category 1 had polyneuropathy/neuropathy, about 10% of the patients in Category 2 and Category 3 reported this comorbidity. The severity of cognitive dysfunction was mild in all patients in Category 1 and in majority (> 70%) of the patients in Category 2 and 3 (Additional file, Table 2).

**Table 2:** Information about options and eligibility for DAT by categories of disease control (MANAGE-PD tool)

		Disease control categorization by MANAGE-PD tool		
		Category 1 (n = 50)	Category 2 (n = 125)	Category 3 (n = 103)
Patient is informed about DAT options, n (%)	Yes	18 (36.0)	65 (52.0)	63 (61.2)
	No	32 (64.0)	60 (48.0)	40 (38.8)
Eligible for DAT (physician’s assessment), n (%)	Yes	6 (12.0)	41 (32.8)	78 (75.7)
	No	44 (88.0)	84 (67.2)	25 (24.3)

Category 1: Currently well controlled, Category 2: Inadequately controlled—might benefit from further oral optimization, Category 3: Inadequately controlled—might benefit from DAT. DAT, device-aided therapy; N/n: number of patients

### Disease burden

**Fig. 1:**
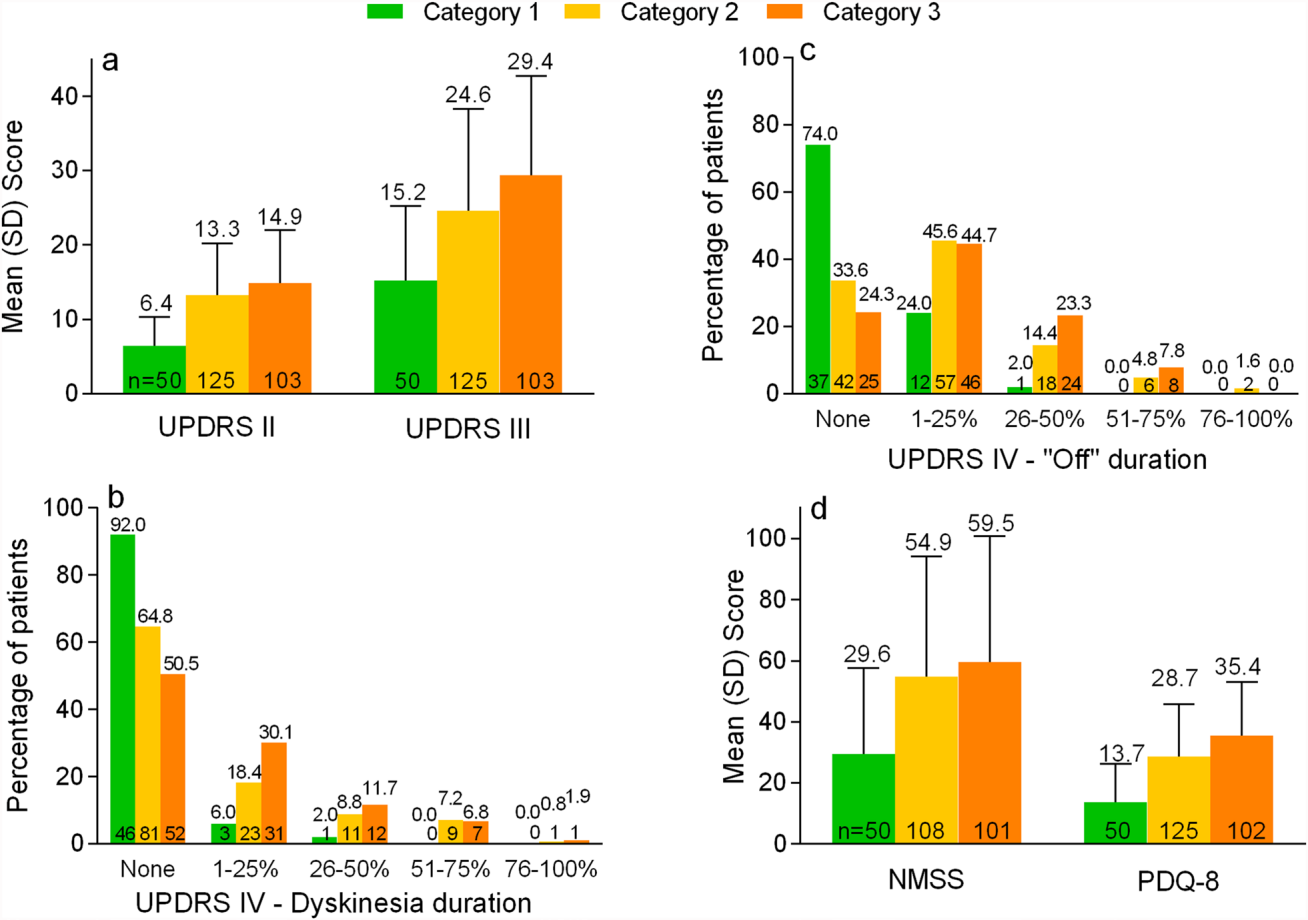
Disease burden in patients by categories of disease control (MANAGE-PD tool). Panels A, D: mean values with standard deviation as whiskers. Panels B and C: percentages. Dyskinesia duration: dyskinesia anytime during the day: at least mildly disabled. “Off” duration: % of the day with “Off” time. The number of patients is shown at the bottom of the bars. NMSS, Non-Motor Symptoms Scale (NMSS); PDQ-8, 8-item Parkinson’s disease Questionnaire; UPDRS, Unified PD Rating Scale (UPDRS parts II-IV)

On average, UPDRS II (activities of daily living) and UPDRS III (motor symptoms) scores were lowest (i.e. lowest disease burden) in patients in Category 1 (UPDRS II: 6.4 ± 3.9, UPDRS III: 15.2 ± 10.1) and somewhat lower for patients in Category 2 (UPDRS II: 13.3 ± 6.9, UPDRS III: 24.6 ± 13.7) compared to patients in Category 3 (UPDRS II: 14.9 ± 7.1, UPDRS III: 29.4 ± 13.3) (Fig. 1A). Motor complications were rather rare in patients in Category 1 (Fig. 1B and C, Additional file, Table 3). Most of these patients had no dyskinesia (92.0%) and no “off” time (74.0%). Dyskinesia was reported by about one third of the patients in Category 2 (dyskinesia anytime during the day: 35.2%, at least mildly disabled: 30.4%) and about half in Category 3 (dyskinesia anytime during the day: 49.5%, at least mildly disabled: 43.7%). The majority of the patients in Category 2 and Category 3 experienced “off” time; most frequently 1–25% of the day (Category 2: 45.6%, Category 3: 44.7%). “Off” time was unpredictable in 10.0% of the patients in Category 1 (Additional file, Table 3), while 37.6% of the patients in Category 2 and 43.7% in Category 3 reported unpredictable “off” time. While no patient in Category 1 experienced sudden “off” time, “off” time occurred suddenly in 20.8% and 25.2% of the patients in Category 2 and Category 3, respectively. The majority of the patients in Category 2 (56.8%) and Category 3 (65.0%) had a Hoehn and Yahr stage ≥ 3 (at least mild to moderate bilateral disease), but only 14.0% of patients in Category 1 were at a stage ≥ 3 (Additional file, Table 3).

According to the severity rating proposed by Ray Chaudhuri et al. [22], a moderate non-motor symptom burden was observed on average in patients in Category 1 (NMSS: 29.6 ± 27.9) but a severe burden was observed in Category 2 (54.9 ± 39.3) and Category 3 (59.5 ± 41.2) (Fig. 1 D). A stronger quality of life impairment was observed in patients with a lower disease control (PDQ-8 for Category 1: 13.7 ± 12.6, Category 2: 28.7 ± 17.2, Category 3: 35.4 ± 17.8).

### Treatment changes

All patients received PD treatment at the study start. Details on current PD treatments are given in Additional file, Table 4.

**Fig. 2:**
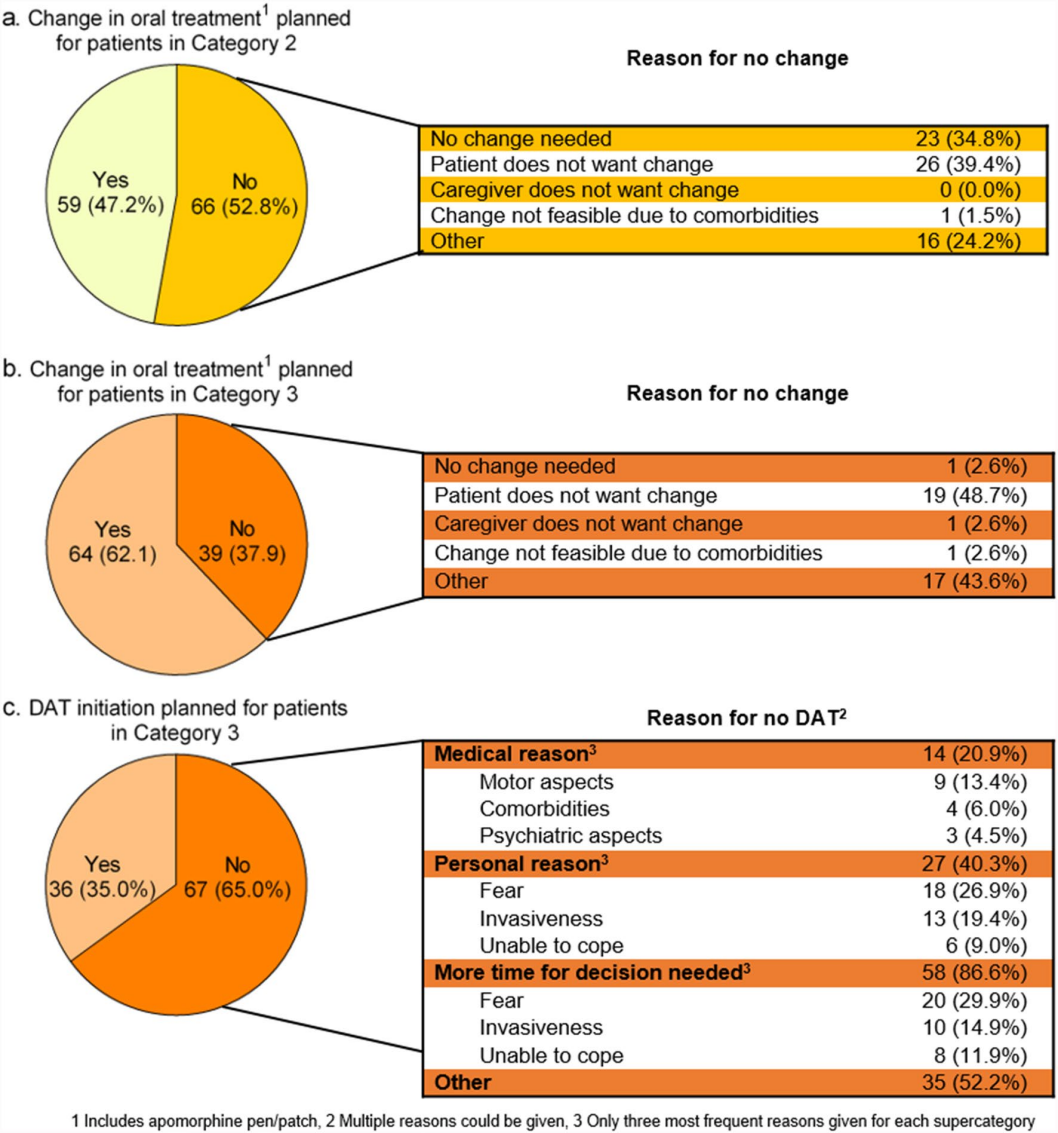
Planned treatment changes and reason for no change in patients in MANAGE-PD tool Category 2 (A) and Category 3 (B). Planned DAT initiation and reason for no initiation in patients in Category 3 (C)

Physicians planned changes in oral treatment for 47.2% of the patients in Category 2 and for 62.1% of the patients in Category 3 (Fig. 2A and 2B). A dose increase was planned for about two-thirds of the patients with an intended treatment change (Category 2: 66.1%, Category 3: 62.5%). Details on planned changes in dosing, frequency, and stopping of oral treatment are given in Additional file, Table 5. In both categories, the most frequent reason for no change in oral treatment was patients’ reluctance towards it. For about one-third of the patients in Category 2, no change was needed according to physicians’ judgment.

Being informed about DAT options was more common in higher categories of MANAGE-PD: 36.0% for Category 1, 52.0% for Category 2 and 61.2% for Category 3 (Table 2).

Similarly, more patients were assessed by physicians as being eligible for DAT in Category 3 (75.7%) than in Categories 2 (32.8%) and 1 (12.0%) (Table 2). According to the logistic regression model, the chance of being considered eligible for DAT was about 3 times higher in patients with troublesome dyskinesia in comparison to those without it (Additional file, Table 6). Similarly, patients with non-motor “off” symptoms had a 2.4–3.4 times higher chance for being eligible for DAT in comparison to patients without these symptoms (Additional file, Table 6).

DAT initiation was planned for 35.0% of the patients in Category 3 (Fig. 2C). Levodopa intestinal gel infusion (47.2%) and deep brain stimulation (41.7%) were the most frequently considered options for these patients (Additional file, Table 7). In 20.9% of the cases, DAT initiation was not planned because of medical reasons. Personal reasons were given by 40.3% of patients for not wanting DAT initiation with fear (26.9%) and invasiveness (19.4%) being most frequently reported. Most patients (86.6%) with no planned DAT initiation needed more time for the decision predominantly because of fear (29.9%) and the invasiveness (14.9%) of the procedure (Fig. 2C). In more than half of the patients (52.2%), there were additional other reasons which were unfortunately not further specified. Details on all reasons are shown in Additional file, Table 8.

## Discussion

This first-reported prospective study utilizing the MANAGE-PD tool provides new evidence supporting its validity. Results showed a clear association between the level of disease control, as determined by MANAGE-PD, and the disease burden, based on patient comorbidities and the scores used to assess motor and non-motor symptoms as well as quality of life. Notably, oral treatment optimization or escalation to DAT were not planned for many patients that were categorized as being inadequately controlled.

The new German PD guidelines provide clear instructions on when DAT should be offered to patients. In patients with levodopa-dependent fluctuations who do not improve sufficiently with oral or transdermal therapy, DAT should be considered. The guidelines state, that the first occurrence of motor fluctuations should be the latest time point to inform patients about DAT options [15]. The guideline recommendations are supported by study data, such as the EARLYSTIM study [24], where neurostimulation proved to be superior to medical therapy alone, even before the appearance of severe disabling motor complications. Similarly, other studies showed that patients benefited from levodopa-carbidopa intestinal gel (LCIG) [3, 4] or subcutaneous infusion of foslevodopa/foscarbidopa (LDp/CDp) [27], independent of their disease duration or age.

In our study, optimization of oral therapy or DAT initiation were not planned for a significant proportion of inadequately controlled patients, i.e., those in MANAGE-PD tool Category 2 or 3. In order to shed light on this discrepancy, and to gain a better understanding of the factors governing the physicians’ decision whether or not to initiate DAT, regression analyses were performed. Based on these analyses, physicians considered patients eligible for DAT initiation more often if the patients experienced motor fluctuations (dyskinesia) and/or non-motor fluctuations (non-motor “off” symptoms). This finding is in line with the recent German treatment guidelines (2023) that suggest considering initiation of DAT when at least one of the following criteria are met: ≥ 5 levodopa intakes per day, ≥ 2 h of “off”-symptoms per day, or ≥ 1 h of troublesome dyskinesia per day. In addition, the importance of non-motor symptoms and non-motor fluctuations is now emphasized [15].

The major obstacle to initiating DAT in our study was not due to disregarding the guideline recommendations, but rather patient reluctance to initiate DAT due to fear and concerns over the invasiveness of the procedure. The high level of patients’ concerns surrounding DAT is strongly supported by additional studies [9, 28]. For instance, in an epidemiological study with 129 PD patients in Italy, 56% of patients reported specifically to be “afraid of advanced treatments” [31].

It must be acknowledged that, despite all efforts, not all specific patient fears can be adequately alleviated by the attending physicians. In addition, our data show that a considerable number of patients had not been provided with information about DAT options (Category 2, 48.0%; Category 3, 38.8%), which would be not in line with the recommendations of the German PD guidelines [15]. The observation that a significant proportion of patients in MANAGE-PD tool Categories 2 and 3 may not have been adequately informed about DAT options is consistent with a previous study that also used the MANAGE-PD tool on DAT-naïve patients. In that study, more than 40% of patients in Category 3 did not report discussions with providers about DATs [12]. It can be speculated that inadequate patient education on DATs may stem from factors such as limited consultation time during neurologist visits, where multiple urgent issues need to be addressed, a lack of awareness regarding the importance of early education, patients’ difficulty in retaining the information provided, and the fact that not all neurologists are thoroughly informed about DATs.

Due to the complexity of the various DAT options, patients would surely benefit from obtaining detailed information material in lay language covering DAT options and various related aspects, such as follow-up care. Patients should receive this information early on in the course of their disease to allow them to process and clarify potential concerns, as has been also proposed by Timpka et al. [32]. The potential benefit of earlier provision of information is supported by the finding in our study that a significant proportion of patients stated that they needed more time to decide regarding initiation of DAT. Additionally, frequent and early utilization of MANAGE-PD or of the “Parkinson Check” self-test (the patient version of MANAGE-PD, Sect. 1) may also support the patients’ decision-making process and autonomy and reduce their mental barriers for treatment optimization. It is worth noting that the results of this study did not indicate that patients’ caregivers were an impediment to initiation of DAT, as “caregiver refusal “ or “lacking support from caregiver” was not documented in any patient. However, the indirect influence of caregiver opinion on patient decisions should not be overlooked.

A contributing factor to patient avoidance of DAT appears to be the high degree of invasiveness of the various treatment options. In the future, the further development of other, less invasive treatment options, such as subcutaneous foslevodopa/foscarbidopa [26], may help alleviate such concerns in hesitant patients, ultimately leading to better disease control.

Apart from the major obstacle of patient’s concerns regarding DAT, it is well established that there is a relatively conservative approach to treatment escalation in Germany. In the Observe-PD study [11, 19], 58.2% of the patients with advanced PD were eligible for DAT, but only 40.8% actually received the treatment. This rate is very similar to our study, where 42.2% of the patients were assessed as having advanced PD (data not shown) and only 35.0% of patients in Category 3 planned to receive DAT. Similarly, in the BALANCE study, it was shown that LCIG treatment in Germany and Switzerland was delayed “beyond the established indication criteria” [34]. Our study results support the data of another study in Germany (Care4PD study [13] where many patients with advanced PD did not receive DAT; among the patients without DAT in that study, > 50% fulfilled at least one advanced PD criterion. The Care4PD study advocates the use of “preselection tools” providing another justification for the use of tools like MANAGE-PD stating: “precise and early identification of advanced PD symptoms (and therapy-resistant tremor should be implemented in future DAT preselection tools and educational trainings” [13].

Our study cohort exhibited a high degree of concordance with epidemiological data on patients with Parkinson's disease, particularly with respect to its mean age and its marked preponderance of male patients. As the mean time since PD diagnosis was 7.4 years, the majority of patients experienced a considerable degree of disability caused by their disease based on the typical time course [25]. Most patients reported having at least one comorbidity and common comorbidities included hypertension, sleep disorders, cardiac abnormalities or cardiovascular disease, and depression. It is worth noting that sleep disorders were already present in many patients early on, already occurring in 20% of patients in Category 1 (20.0%), which illustrates the high prevalence of this symptom throughout the course of the disease [5, 7]. Notably, there was also a relatively high incidence of polyneuropathy in patients in Category 2 and 3 (altogether approximately 10%), which serves to highlight the importance of careful monitoring of vitamin B12 levels during levodopa therapy [6, 33].

### Limitations

This study has the inherent shortcomings of any non-interventional study and its various risks of bias. Selection bias may have occurred as sites and patients participated based on convenience. Thus, only the subset of willing and motivated sites and patients were selected. Moreover, the validity of the data may be limited due to a relatively small overall sample size, which was further reduced by the separate analysis of the three different categories. Lastly, because patients were recruited in Germany only, these findings may not be generalizable to an international patient cohort.

Upon closer examination of the clinical scores between the categories, it is evident that there were considerable differences between Category 1 and 2, but comparatively smaller differences between Category 2 and 3. Consequently, there were many patients in Category 2 who were actually very close to being directly eligible for a DAT based on their disease burden. This observation was confirmed by Antonini et al. [1] who also reported larger differences between Category 1 and 2 than those between Category 2 and 3. Regarding the ability to differentiate between the categories, the MANAGE-PD tool might benefit from some further refinement to better distinguish and separate those patients that may still benefit from additional oral therapy optimization and those who should be directly offered DAT. A plausible refinement approach would involve assigning greater weight to motor fluctuations (frequency of troublesome dyskinesia) and non-motor fluctuations (frequency of non-motor "off" symptoms) that correlated with physicians' perceptions of patient eligibility for DAT in our linear regression model. However, in real life, the difference between these two groups may be rather minuscule and also depend on physician’s preference. As a progressive disease, PD ultimately tends to reach a stage where further oral optimization brings only minimal benefits. Finally, it must be considered that any additional refinements to the MANAGE-PD tool may compromise the highly valued quality of simplicity that makes the tool so useful.

## Conclusions

In summary, this study provides new prospective evidence supporting the validity of the MANAGE-PD tool, as the tool categorization was strongly associated with the burden of PD in all the clinical scales used and with increased comorbidity burden. Non-motor “off” symptoms and troublesome dyskinesia were important indicators for being considered eligible for DAT. The study highlights the importance of providing patients with timely necessary information about the benefits and risks of DATs to address their concerns and to enable them to make informed decisions about DAT initiation when it is offered. In short, the results of this work highlight the benefits of using the MANAGE-PD tool more widely in routine practice.

## Supplementary Information

Additional file 1.

## Data Availability

AbbVie is committed to responsible data sharing regarding the clinical trials we sponsor. This includes access to anonymized, individual, and trial-level data (analysis data sets), as well as other information (e.g., protocols, clinical study reports, or analysis plans), as long as the trials are not part of an ongoing or planned regulatory submission. This includes requests for clinical trial data for unlicensed products and indications. These clinical trial data can be requested by any qualified researchers who engage in rigorous, independent, scientific research, and will be provided following review and approval of a research proposal, Statistical Analysis Plan (SAP), and execution of a Data Sharing Agreement (DSA). Data requests can be submitted at any time after approval in the US and Europe and after acceptance of this manuscript for publication. The data will be accessible for 12 months, with possible extensions considered. For more information on the process or to submit a request, visit the following link https://vivli.org/ourmember/abbvie/ then select “Home”.

## Abbreviations

DAT: Device-aided therapy
DBS: Deep brain stimulation
eCRF: Electronic case report form
MANAGE-PD: Making informed decision to aid timely management of Parkinson’s disease
MAO: Monoamine oxidase
LCIG: Levodopa-carbidopa intestinal gel
LDp/CDp: Foslevodopa/foscarbidopa
NMSS: Non-motor symptom assessment scale for Parkinson’s disease
PD: Parkinson’s disease
PDQ-8: Parkinson’s disease quality of life questionnaire
UPDRS: Unified Parkinson’s disease rating scale
