# Supplementary material for: Real-world disease burden and planned treatment optimization after MANAGE-PD implementation in Germany: a cross-sectional study
Source: Neurol Res Pract. 2025 May 12;7(1):31. doi: 10.1186/s42466-025-00383-2 (PMC12067699; doi:10.1186/s42466-025-00383-2)
Supplement: Supplementary file 1 — Additional file 1. [file 42466_2025_383_MOESM1_ESM.docx]

**ADDITIONAL FILE**

**Real-world disease burden and planned treatment optimization after MANAGE PD implementation in Germany**

Prof. Martin Südmeyer^1,2^, Prof. David J. Pedrosa^3^, Dr. Frank Siebecker^4^, Dr. Carolin Arlt^5^, Dr. Jaakko Kopra^6^, Prof. Wolfgang H. Jost^7^

**Table 1.** PD- and non-PD-related comorbidities

|  | **Disease control categorization by**  **MANAGE PD tool** | | |  |
| --- | --- | --- | --- | --- |
|  | **Category 1**  (n=50) | **Category 2**  (n=125) | **Category 3**  (n=103) | **Total**  (N=278) |
| At least one comorbidity, n (%) | 36 (72.0) | 111 (88.8) | 93 (90.3) | 240 (86.3) |
| Orthostatism | 4 (8.0) | 9 (7.2) | 15 (14.6) | 28 (10.1) |
| Cardiac abnormalities/ Cardiovascular disease | 12 (24.0) | 24 (19.2) | 28 (27.2) | 64 (23.0) |
| Polyneuropathy/Neuropathy | 1 (2.0) | 12 (9.6) | 11 (10.7) | 24 (8.6) |
| Depression | 5 (10.0) | 26 (20.8) | 28 (27.2) | 59 (21.2) |
| Cognitive dysfunction | 4 (8.0) | 22 (17.6) | 25 (24.3) | 51 (18.3) |
| Sleep disorders | 10 (20.0) | 32 (25.6) | 31 (30.1) | 73 (26.3) |
| Fatigue | 2 (4.0) | 6 (4.8) | 13 (12.6) | 21 (7.6) |
| Hypertension | 18 (36.0) | 43 (34.4) | 46 (44.7) | 107 (38.5) |
| Diabetes mellitus | 4 (8.0) | 9 (7.2) | 9 (8.7) | 22 (7.9) |
| Any malignancy | 3 (6.0) | 14 (11.2) | 10 (9.7) | 27 (9.7) |
| Other | 15 (30.0) | 47 (37.6) | 35 (34.0) | 97 (34.9) |
| Category 1: Currently well controlled, Category 2: Inadequately controlled – might benefit from further oral optimization, Category 3: Inadequately controlled – might benefit from DAT, N/n: number of patients | | | | |

**Table 2.** Severity of cognitive dysfunction

|  | **Disease control categorization by MANAGE PD tool** | | |  |
| --- | --- | --- | --- | --- |
| **Severity** | **Category 1**  (n=50) | **Category 2**  (n=125) | **Category 3**  (n=103) | **Total**  (N=278) |
| Mild | 4 (100.0) | 16 (72.7) | 22 (88.0) | 42 (82.4) |
| Moderate | 0 (0.0) | 4 (18.2) | 3 (12.0) | 7 (13.7) |
| Severe/dementia | 0 (0.0) | 2 (9.1) | 0 (0.0) | 2 (3.9) |
| Category 1: Currently well controlled, Category 2: Inadequately controlled – might benefit from further oral optimization, Category 3: Inadequately controlled – might benefit from DAT, N/n: number of patients | | | | |

**Table 3.** UPDRV IV scores and modified Hoehn and Yahr stage

|  | | **Disease control categorization by MANAGE PD tool** | | |  |
| --- | --- | --- | --- | --- | --- |
|  | | **Category 1**  (n=50) | **Category 2**  (n=125) | **Category 3**  (n=103) | **Total**  (N=278) |
| Dyskinesias - disability | Not disabling | 49 (98.0) | 87 (69.6) | 58 (56.3) | 194 (69.8) |
|  | Mildly | 1 (2.0) | 15 (12.0) | 17 (16.5) | 33 (11.9) |
|  | Moderately | 0 (0.0) | 14 (11.2) | 16 (15.5) | 30 (10.8) |
|  | Severely | 0 (0.0) | 8 (6.4) | 10 (9.7) | 18 (6.5) |
|  | Completely | 0 (0.0) | 1 (0.8) | 2 (1.9) | 3 (1.1) |
| “Offs” - unpredictable | Yes | 5 (10.0) | 47 (37.6) | 45 (43.7) | 97 (34.9) |
|  | No | 45 (90.0) | 78 (62.4) | 58 (56.3) | 181 (65.1) |
| “Offs” - sudden | Yes | 0 (0.0) | 26 (20.8) | 26 (25.2) | 52 (18.7) |
|  | No | 50 (100.0) | 99 (79.2) | 77 (74.8) | 226 (81.3) |
| Modified Hoehn and Yahr stage | Stage 0 | 1 (2.0) | 0 (0.0) | 0 (0.0) | 1 (0.4) |
|  | Stage 1 | 11 (22.0) | 4 (3.2) | 1 (1.0) | 16 (5.8) |
|  | Stage 1.5 | 13 (26.0) | 11 (8.8) | 2 (1.9) | 26 (9.4) |
|  | Stage 2 | 13 (26.0) | 16 (12.8) | 19 (18.4) | 48 (17.3) |
|  | Stage 2.5 | 5 (10.0) | 23 (18.4) | 14 (13.6) | 42 (15.1) |
|  | Stage 3 | 7 (14.0) | 55 (44.0) | 48 (46.6) | 110 (39.6) |
|  | Stage 4 | 0 (0.0) | 15 (12.0) | 14 (13.6) | 29 (10.4) |
|  | Stage 5 | 0 (0.0) | 1 (0.8) | 5 (4.9) | 6 (2.2) |
| Category 1: Currently well controlled, Category 2: Inadequately controlled – might benefit from further oral optimization, Category 3: Inadequately controlled – might benefit from DAT, Stage 0: No signs of disease, Stage 1: Unilateral disease, Stage 1.5: Unilateral plus axial involvement, Stage 2 Bilateral disease, without impairment of balance, Stage 2.5: Mild bilateral disease, with recovery on pull test, Stage 3: Mild to moderate bilateral disease; some postural instability; physically independent, Stage 4: Severe disability; still able to walk or stand unassisted, Stage 5: Wheelchair bound or bedridden unless aided, N/n: number of patients | | | | | |

**Table 4.** Current PD treatments (at study inclusion)

|  | **Disease control categorization by MANAGE PD tool** | | |  |
| --- | --- | --- | --- | --- |
|  | **Category 1**  (n=50) | **Category 2**  (n=125) | **Category 3**  (n=103) | **Total**  (N=278) |
| Any current PD treatment, n (%) | 50 (100.0) | 125 (100.0) | 103 (100.0) | 278 (100.0) |
| Oral levodopa | 50 (100.0) | 125 (100.0) | 103 (100.0) | 278 (100.0) |
| Oral dopamine agonist(s) | 22 (44.0) | 59 (47.2) | 50 (48.5) | 131 (47.1) |
| Dopamine agonist patch | 4 (8.0) | 22 (17.6) | 14 (13.6) | 40 (14.4) |
| Apomorphine s.c. as rescue injection/pen | 0 (0.0) | 2 (1.6) | 2 (1.9) | 4 (1.4) |
| Catechol-o-methyltransferase (COMT) inhibitors | 6 (12.0) | 40 (32.0) | 43 (41.7) | 89 (32.0) |
| Monoamine oxidase B (MAO-B) inhibitors | 18 (36.0) | 43 (34.4) | 37 (35.9) | 98 (35.3) |
| Amantadine | 5 (10.0) | 22 (17.6) | 18 (17.5) | 45 (16.2) |
| Other | 4 (8.0) | 9 (7.2) | 13 (12.6) | 26 (9.4) |
| Category 1: Currently well controlled, Category 2: Inadequately controlled – might benefit from further oral optimization, Category 3: Inadequately controlled – might benefit from DAT, N/n: number of patients | | | | |

**Table 5.** Details on changes for patients in MANAGE-PD tool Category 2 and Category 3 with planned change in PD oral treatment

|  |  | **Disease control categorization by MANAGE PD tool** | |  |
| --- | --- | --- | --- | --- |
|  |  | **Category 2**  (n=59) | **Category 3**  (n=64) | **Total**  (n=123) |
| Dosing, n (%) | Increase | 39 (66.1) | 40 (62.5) | 79 (64.2) |
|  | Decrease | 12 (20.3) | 15 (23.4) | 27 (22.0) |
|  | No change/Unknown | 8 (13.6) | 9 (14.1) | 17 (13.8) |
| Frequency, n (%) | Increase | 23 (39.0) | 24 (37.5) | 47 (38.2) |
|  | Decrease | 3 (5.1) | 8 (12.5) | 11 (8.9) |
|  | No change/Unknown | 33 (55.9) | 32 (50.0) | 65 (52.8) |
| Stop of oral treatment, n (%) | Yes | 1 (1.7) | 10 (15.6) | 11 (8.9) |
|  | No/Unknown | 58 (98.3) | 54 (84.4) | 112 (91.1) |

Category 1: Currently well controlled, Category 2: Inadequately controlled – might benefit from further oral optimization, Category 3: Inadequately controlled – might benefit from DAT, n: number of patients

**Table 6.** Logistic regression model for patient eligibility for DAT in MANAGE-PD tool Category 2 and 3.

| **Symptom** | | **OR** | **95% CI** | **p-value** |
| --- | --- | --- | --- | --- |
| Frequency of troublesome dyskinesia (vs. Never) | | | | |
|  | Rarely (≤ 1 per week) | 2.9 | 0.5; 15.9 | 0.2213 |
|  | Some of the time (several times per week) | 2.8 | 1.2; 6.7 | 0.0183 |
|  | Most/All of the time (daily) | 2.8 | 1.2; 6.5 | 0.0136 |
| Frequency of non-motor “off” symptoms (vs. Never) | | | | |
|  | Rarely (≤ 1 per week) | 2.4 | 1.1; 5.4 | 0.0363 |
|  | Some of the time (several times per week) | 2.8 | 1.3; 5.9 | 0.0067 |
|  | Most/All of the time (daily) | 3.4 | 1.5; 7.9 | 0.0034 |
| CI: confidence interval, OR: odds ratio, Reference category for each variable: Never | | | | |

**Table 7.** Type of DAT in patients in MANAGE-PD tool Category 3 and a planned DAT initiation

| **Type of initiated DAT therapy,** n (%) | **Patients with planned DAT initiation** (n=36) |
| --- | --- |
| Deep brain stimulation (DBS) | 15 (41.7) |
| Continuous apomorphine s.c. infusion | 1 (2.8) |
| Levodopa intestinal gel | 17 (47.2) |
| Other | 3 (8.3) |

**Table 8.** Reasons for no DAT initiation in patients in MANAGE-PD tool Category 3

| **Reason for not initiating DAT**, n (%) | | **Patients with no planned DAT initiation** (n=67) |
| --- | --- | --- |
| **Medically not eligible** | | **14 (20.9)** |
|  | Age | 2 (3.0) |
|  | Cognitive aspects | 2 (3.0) |
|  | Psychiatric aspects | 3 (4.5) |
|  | Motor aspects | 9 (13.4) |
|  | Comorbidities | 4 (6.0) |
| **Patient’s personal reasons** | | **27 (40.3)** |
|  | Weight of the pump | 3 (4.5) |
|  | Size of the pump | 4 (6.0) |
|  | Stigma | 5 (7.5) |
|  | Invasiveness | 13 (19.4) |
|  | Fear | 18 (26.9) |
|  | Lacking support from caregiver | 0 (0.0) |
|  | Caregiver refusal | 0 (0.0) |
|  | Unable to cope | 6 (9.0) |
| **Patient needs more time to decide** | | **58 (86.6)** |
|  | Weight of the pump | 1 (1.5) |
|  | Size of the pump | 2 (3.0) |
|  | Stigma | 4 (6.0) |
|  | Invasiveness | 10 (14.9) |
|  | Fear | 20 (29.9) |
|  | Lacking support from caregiver | 0 (0.0) |
|  | Caregiver refusal | 0 (0.0) |
|  | Unable to cope | 8 (11.9) |
| **Cost/reimbursement** | | **0 (0.0)** |
| **Other** | | **35 (52.2)** |
| Multiple entries possible | | |
